# Demethyleneberberine alleviated the inflammatory response by targeting MD-2 to inhibit the TLR4 signaling

**DOI:** 10.3389/fimmu.2023.1130404

**Published:** 2023-04-24

**Authors:** Yaxing Zhao, Peng Liu, Haofan Luan, Hua Jiang, Yingmei Xu, Yuanqiang Zhang, Yubin Zhang, Ruiyan Li

**Affiliations:** ^1^ State Key Laboratory of Natural Medicines, Department of Biochemistry, China Pharmaceutical University, Nanjing, China; ^2^ Shanghai Tenth People’s Hospital, School of Medicine, Tongji University, Shanghai, China; ^3^ Key Laboratory of Carbohydrate Chemistry and Biotechnology, Ministry of Education, School of Pharmaceutical Sciences, Jiangnan University, Wuxi, China

**Keywords:** demethyleneberberine, myeloid differentiation protein-2, TLR4 signaling, inflammatory response, TNBS-induced colitis

## Abstract

**Introduction:**

The colitis induced by trinitrobenzenesulfonic acid (TNBS) is a chronic and systemic inflammatory disease that leads to intestinal barrier dysfunction and autoimmunedisorders. However, the existing treatments of colitis are associated with poor outcomes, and the current strategies remain deep and long-time remission and the prevention of complications. Recently, demethyleneberberine (DMB) has been reported to be a potential candidate for the treatment of inflammatory response that relied on multiple pharmacological activities, including anti-oxidation and antiinflammation. However, the target and potential mechanism of DMB in inflammatory response have not been fully elucidated.

**Methods:**

This study employed a TNBS-induced colitis model and acute sepsis mice to screen and identify the potential targets and molecular mechanisms of DMB *in vitro* and *in vivo*. The purity and structure of DMB were quantitatively analyzed by high-performance liquid chromatography (HPLC), mass spectrometry (MS), Hydrogen nuclear magnetic resonance spectroscopy (^1^H-NMR), and infrared spectroscopy (IR), respectively. The rats were induced by a rubber hose inserted approximately 8 cm through their anus to be injected with TNBS. Acute sepsis was induced by injection with LPS *via* the tail vein for 60 h. These animals with inflammation were orally administrated with DMB, berberine (BBR), or curcumin (Curc), respectively. The eukaryotic and prokaryotic expression system of myeloid differentiation protein-2 (MD-2) and its mutants were used to evaluate the target of DMB in inflammatory response.

**Resluts:**

DMB had two free phenolic hydroxyl groups, and the purity exceeded 99% in HPLC. DMB alleviated colitis and suppressed the activation of TLR4 signaling in TNBS-induced colitis rats and LPS-induced RAW264.7 cells. DMB significantly blocked TLR4 signaling in both an MyD88-dependent and an MyD88-independent manner by embedding into the hydrophobic pocket of the MD-2 protein with non-covalent bonding to phenylalanine at position 76 in a pi–pi T-shaped interaction. DMB rescued mice from sepsis shock induced by LPS through targeting the TLR4–MD-2 complex.

**Conclusion:**

Taken together, DMB is a promising inhibitor of the MD-2 protein to suppress the hyperactivated TLR4 signaling in inflammatory response.

## Introduction

The colitis induced by TNBS is a chronic and systemic inflammatory disease that leads to intestinal barrier dysfunction and autoimmune disorders; it might come from many risk factor interactions between genetic susceptibility, diet, immune response, and gut microbiota (1). The current strategies in the treatment of colitis are deep and long-time remission and the prevention of complications (2, 3). However, the therapeutics of colitis are not enough for the treatment of high-risk patients (4). Disorders of the innate and adaptive immune responses in intestinal mucosa exacerbated the inflammatory response in colitis patients (5). Insight into the pathogenesis of colitis about inflammatory and immune response could offer novel targets, potential therapies, and promising agents for the treatment of colitis.

Myeloid differentiation protein-2 (MD-2) is an important co-receptor component of toll-like receptor 4 (TLR4) (6) and provides a hydrophobic pocket through two anti-parallel β-sheets interacting with lipid A of lipopolysaccharide (LPS) and mediates the downstream cascade (7). TLR4 acts as the first line of defense, recognizes the pathogen-associated molecular pattern (PAMP), and provides stimulus signals to the host to activate the innate immune system. LPS is captured by the LPS-binding protein and transferred to CD14 and MD-2 (8). When MD-2 combines with LPS, the TLR4–MD-2 complex is triggered to form a heterodimer, which leads to recruiting two major adaptors: MyD88 and TRIF (6, 9). Finally, the intracellular NF-κB signaling is activated to produce pro-inflammatory cytokines, such as IL-1β, TNF-α, and IFN-α (10).

The dynamic equilibrium between commensal bacteria and host immune response is crucial in the development of colitis (11). Given the high concentration of LPS in gastrointestinal lumen, it is important for the host to maintain high sensitivity to intestinal pathogens and tolerance toward commensal bacteria. However, upregulated MD-2 expression is common in intestinal inflammatory response (12). MD-2 has two different binding sites for LPS and TLR4, respectively; the Phe119–Lys132 residues are combined with LPS, and the Cys95–Cys105 residues bonded directly to TLR4 (13). MD-2 lacks transmembrane and intracellular domains, but possesses a similar affinity to LPS in the absence or presence of TLR4 (14). A previous study demonstrated that LPS hyposensitivity is reversed by the overexpression of MD-2 or an additional soluble MD-2 protein, and MD-2^−/−^ mice significantly survived LPS shocking (15). Therefore, MD-2 is selected as a potential target for LPS infection.

Demethylenberberine (DMB) is an isoquinoline alkloid with two free ortho phenolic hydroxyl groups (16). It is the effective component of Chinese traditional medicine *Coptis chinensis* Franch. Our previous study indicated that DMB was a natural mitochondrion-targeting antioxidant agent with cationic properties (17) that alleviated colitis in mice through regulating NF-κB signaling (18). However, the target of DMB in the treatment of inflammatory response is still not elucidated clearly. In this study, the target of DMB was anchored to the MD-2 protein, and the interactions between DMB and MD-2 were explored. In addition, we evaluated the potential of the MD-2 protein as the therapeutic target in inflammatory response.

## Materials and methods

### Reagents

Trinitrobenzenesulfonic acid (TNBS) was purchased from Solarbio (Beijing, China). LPS was purchased from Sigma-Aldrich (Missouri, USA). Antibodies against IκB (1:1,000, Cat #9242), p-IκB (1:1,000, Cat #2859), IRF3 (1:1,000, Cat #4302), and TRAF6 (1:1,000, Cat #67591) were purchased from Cell Signaling Technology (Danvers, Colorado, USA). Antibodies against IL-1β (1:1,000, Cat AF4006), MyD88 (1:1,000, Cat DF6162), and GAPDH (1:5,000, Cat T0004) were purchased from Affinity Biosciences Ltd. (liyang, China). MD-2 antibody (1:1,000, Cat BS7308) was obtained from Biogot Technology Co., Ltd (Nanjing, China). Berberine and Curc were obtained from Meilunbio (Dalian, China).

### Preparation and qualitative analysis of DMB

The preparation of DMB was performed in accordance with our previous report (17). The purity and structure of DMB were quantitatively analyzed by high-performance liquid chromatography (HPLC), mass spectrometry (MS), ^1^H-NMR, and infrared spectroscopy (IR).

### Animals

Male Sprague Dawley rats (8 weeks, 180–200 g) and female C57BL/6 mice (8 weeks, 18–22 g) were purchased from Changzhou Cavens Experimental Animal Limited Company (Changzhou, China). All animal care procedures in this study were carried out according to the Guide for the Care and Use of Laboratory Animals (Ministry of Science and Technology of China, 2006) and approved by the related ethical regulations of China Pharmaceutical University. The mice were housed as previously reported (19, 20).

### Colitis induced by TNBS

Rats were randomly divided into five groups: CON, TNBS, L-DMB, H-DMB, and BBR (*n* = 8). Rats in the CON and TNBS groups were gavaged with 0.5% CMC-Na solution once a day; the other groups (L-DMB: 100 mg/kg/day, H-DMB: 200 mg/kg/day, and BBR: 200 mg/kg/day) were separately gavaged with DMB or BBR. All rats were fasted for 40 h until modeling and anesthetized by intraperitoneal injection of 10% chloral hydrate (3.5 μl/g body weight). TNBS solution (4 mg in 1 ml of 25% ethanol aqueous solution, 4 μl/g body weight) was administered into the colonic lumen *via* a rubber hose inserted approximately 8 cm through the anus of the rat. Correspondingly, rats in the CON group were only administered with a water solution. After modeling, all rats continued to be treated for 7 days. The disease activity index (DAI) scores were determined on the last day, and the DAI scoring criteria were as previously reported (19). On day 21, rats were sacrificed using isoflurane anesthesia and colonic tissue was removed for further studies.

### Induction of acute sepsis

Female mice were randomly divided into CON, LPS, DMB, BBR, and Curc groups (*n* = 6). Mice in the CON and LPS groups were gavaged with 0.5% CMC-Na solution once a day, and the other groups were gavaged for 7 days. On day 8, all mice received drugs or vehicle prevention for 2 h and injected with LPS (20 mg/kg) *via* the tail vein; meanwhile, the mice in the CON group were injected with saline. Mouse status and death were recorded every 2 h. Colonic tissues were collected for later experiments.

### Cell lines and plasmid construction

RAW264.7 cells were cultured in a 37°C, 5% CO_2_ incubator and covered with high-sugar DMEM media containing 10% (v/v) Fetal Bovine Serum (FBS), 100 U/ml penicillin G, and 100 μg/ml streptomycin.

### Eukaryotic overexpression plasmid construction

MD-2 gene (GenBank ID: 17087) and TLR4 gene (GenBank ID: 21898) were amplified by polymerase chain reaction (PCR) from cDNA of RAW264.7 cells; the primer sequences and restriction endonucleases are shown in **Table 1**. The overexpression plasmids of pcDNA3.1(+)-MD-2 and pcDNA3.1(+)-TLR4 were transfected into RAW264.7 cells through a liposomal transfection reagent with free serum and antibiotic for 24 h, then changed to a normal medium with 10 μM DMB and continued to culture for 12 h; afterwards, 100 ng/ml LPS was added to stimulate the cells for 6 h. Protein and mRNA were extracted from the cells.

**Table 1:** The primer sequences for plasmid construction.

Genes	Primer	Sequences (5’-3’)	Restriction endonuclease
Eukaryotic MD-2	Fwd	CGGGATCCGCCACCATGTTGCCATTTATTCTCTTTTC	BamH I Xho I
	Rev	CGCTCGAGCTAATTGACATCACGGCGGTGAATG	
Eukaryotic TLR4	Fwd	CGGGATCCGCCACCATGATGCCTCCCTGGCTCCTGGCTAGG	BamH I Not I
	Rev	CGGCGGCCGCTCAGGTCCAAGTTGCCGTTTCTTGTTCTTCC	
Prokaryotic MD-2	Fwd	CGGGATCCATGTTGCCATTTATTCTCTTTTCGACGC	BamH I Xho I
	Rev	CGCTCGAGCTAATTGACATCACGGCGGTGAATG	
MD-2 ^W23A^	Fwd	GCAGAATGCCTGTTGCTTCTCAGATTCAGTCAATATGGGAG	
	Rev	CAACAGGCATTCTGCAACTCCTCCGATGCAATTATTTCCTA	
MD-2 ^F76A^	Fwd	TAGGTTTGCATATAAATATTTTAAGTTTCCTCTTGGAATGA	
	Rev	TTATATGCAAACCTATTCATCAGTGTCAACTCCATAGAGTT	
MD-2 ^G97A^	Fwd	ATCATGTGCATGGCACAGAACTTCCTTACGCTTCGGCAAC	
	Rev	TGCCATGCACATGATGATGACTATTCTTTTTGCAGAGCTCT	
MD-2 ^Y102A^	Fwd	AAAAGATGCGTCATCATCATGTCCATGGCACAGAACTTCC	
	Rev	GATGACGCATCTTTTTGCAGAGCTCTGAAAGGAGAGACTG	

### Prokaryotic plasmid construction and protein renaturation

The recombinant *E. coli BL21*, carrying pET-28a (+)-MD-2, was induced by 0.5 mM IPTG at 37°C, with rotary shaking at 200 rpm for 6 h. The bacteria were ultrasonically broken (60% power, ultrasonic for 3 s, intermittent for 3 s, with a total time of 30 min) and centrifuged at 12,000 rpm for 20 min. The inclusion body was enriched and collected by a nickel column–His-tag system from bacterial lysis supernatant. The inclusion body was refolded at 4°C overnight *via* an oxidized and reduced glutathione solution, and dialyzed four times to remove excessive salt in a 0.01 M PBS solution at 4°C for 2 h. Finally, the MD-2 protein solution was lyophilized and dissolved into the 0.01 M PBS solution.

### Molecular docking

Molecular docking of DMB and the target protein (MD-2) was performed using Schrödinger Maestro software suite (version 9.1,Schrödinger, L.L.C. American) and Molecular Operating Environment (MOE) software. The Protein Data Bank (PDB) database was utilized to download the MD-2 protein structures (http://www.rcsb.org/). The MD-2 protein structure was processed by Schrodinger software and finally saved as a receptor. Finally, using default software parameters, DMB was docked with the MD-2 protein by flexible docking (21).

### Construction and purification of MD-2 mutations

Four mutants of MD-2 (MD-2^W23A^, MD-2^F76A^, MD-2^G97A^, and MD-2^Y102A^) were constructed by site-directed mutagenesis technology, and the primers involved in mutation are shown in **Table 1**. All plasmids were transfected into *E. coli BL21* and verified by gene sequencing. The mutants of MD-2 were extracted according to the previously described scheme. Meanwhile, the eukaryotic overexpression plasmids of pcDNA3.1(+)-MD-2 were also constructed and transfected into RAW264.7 cells.

### Construction of plasmids expressing MD-2-specific shRNA

Two shRNA sequences against the mouse MD-2 were downloaded from Sigma-Aldrich Trading Co., Ltd. (Darmstadt, Germany). The interference sequences are shown in **Table 2**. All interference sequences were loaded into the *pLVTHM* plasmid. The plasmids were transferred into RAW264.7 cells with free serum and antibiotic for 48 h, then changed to a normal medium with 10 μM DMB and continued to culture for 12 h; afterwards, 100 ng/ml LPS was added to stimulate the cells for 6 h to collect and extract protein.

**Table 2:** The sequences of shMD-2 and shTLR4.

Genes	Sequence (5’-3’)
shMD-2-1	GAAGCAACAGTGGTTCTGCAA
shMD-2-2	CAGTGTCAACTCCATAGAGTT

### Ultraviolet absorption spectroscopy of DMB

All the measurements were performed in a 96-well standard microplate. Briefly, the ultraviolet (UV) absorption of different concentrations of DMB and MD-2 was mixed in PBS (pH 7.4) and evaluated in the range of 230–500 nm after incubating for 60 min at 37°C.

### Fluorescence emission of the MD-2 protein

All fluorescence measurements were done at 37°C in a 96-well standard black microplate. A wavelength of 280 nm was used to excite MD-2 and MD-2 variants. Fluorescence emission was detected in the range of 305–495 nm and emission spectrum was drawn. Briefly, different concentrations of MD-2 protein fluorescence and DMB autofluorescence were detected. The changes in fluorescence emission intensity of MD-2 were measured after premixing with DMB according to different molar ratios of 1:1–1:3 at 37°C for 1 h.

### Western blotting assay

According to the experimental operation process (18), the expressions of MD-2, IκB, p-IκB, IL-1β, MyD88, TRAF6, and IRF3 were detected; GAPDH served as reference.

### Real-time quantitative-PCR assays

The gene expression levels were detected according to our previous report (22). The primer sequences used for qRT-PCR are listed in **Table 3**.

**Table 3:** The sequences for RT-qPCR.

Genes	Forward (5’-3’)	Reverse (5’-3’)	Length
mTLR4	GATGACATTCCTTCTTCAACC	GAGGCCAATTTTGTCTCCACA	233 bp
mMD-2	CGCTGCTTTCTCCCATATTGA	CCTCAGTCTTATGCAGGGTTCA	139 bp
mIL-1β	TGGACCTTCCAGGATGAGGAC	GTTCATCTCGGAGCCTGTAGTG	153 bp
mTNF-α	GGTGCCTATGTCTCAGCCTCTT	GCCATAGAACTGATGAGAGGG	138 bp
mIFN-α	GCCACGGCACAGTCATTGA	TGCTGATGGCCTGATTGTCTT	200 bp
rTNF-α	CAAGCGGAGGAGCAGCTGGAG	GAGGCTGACTTTCTCCTGGTATG	215 bp
rIL-1β	CCAAGCACCTTCTTTTCCTTC	CCAAGCACCTTCTTTTCCTTC	201 bp
rIFN-α	CTCTCCCCTGTCTCATGCCTG	GACCACTCAGCTGCTGCTGGAG	216 bp
mGAPDH	AGGTCGGTGTGAACGGATTTG	TGTAGACCATGTAGTTGAGGTC	122 bp
rGAPDH	CAGGAGCGAGATCCCGCTAAC	CTTGAGGGAGTTGTCATATTTC	203 bp

m, mouse; r, rat.

### Histological study

Hematoxylin-Eosin staining (H&E) assay of colonic tissue was performed according to our previous report (23). The injury inflammation score of colonic tissue was evaluated according to the scoring criteria in **Table 4**.

**Table 4:** Scoring criteria for histological injury of colonic tissue.

Inflammation	Depth of lesion	Crypt destruction	Lesion range(η/%)	Score
None	None	None	0	0
Mild	Submucosa	1/3 Crypt destruction	1–25	1
Severe	Muscularis	2/3 Crypt destruction	26–50	2
	Serous layer	all	51–75	3
			76–100	4

### Statistical analysis

All statistical analysis was performed using GraphPad Prism version 8.0.1 (GraphPad software, San Diego, CA, USA). All data were shown as means ± standard deviation (SD). The Shapiro–Wilk test was used to assess the normality and homogeneity of the results. One-way analysis of variance (ANOVA) and two-way ANOVA followed by Tukey multiple comparisons test were conducted using GraphPad Prism.

## Results

### Quality control of DMB

**Figure 1:**
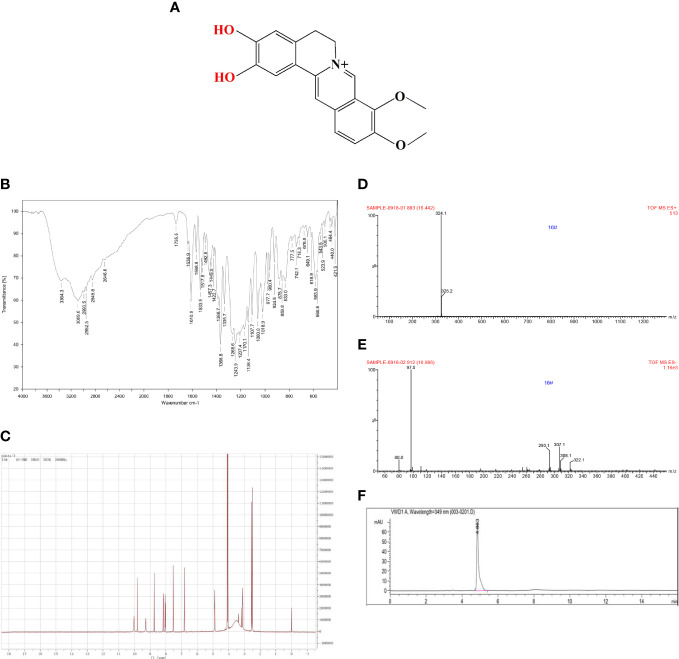
Quality control of DMB. **(A)** The structure of DMB. **(B)** The IR spectrum of DMB. **(C)** The ^1^H-NMR spectrum of DMB. **(D, E)** The MS spectrum of DMB. **(F)** The specific fingerprint spectrum of DMB by HPLC.

As shown in **Figure 1A**, DMB had two free phenolic hydroxyl groups. It was marked with two IR absorption wavelengths at 3,364.3 and 3,089.6 cm^−1^ (**Figure 1B**), and displayed d: 9.83 (s,1H, –OH) and 10.05 (s, 1H, –OH) in ^1^H-NMR (DMSO-d6, 300 MHz) (**Figure 1C**). Electro Spray Ionization-Mass Spectroscopy (ESI-MS) showed a molecular ion peak at m/z 324.1 [M+H]+ (**Figures 1D, E**), and the purity of DMB exceeded 99% in HPLC (**Figure 1F**). These results showed that DMB can be used in subsequent experiments.

### DMB relieved colitis rats induced by TNBS

**Figure 2:**
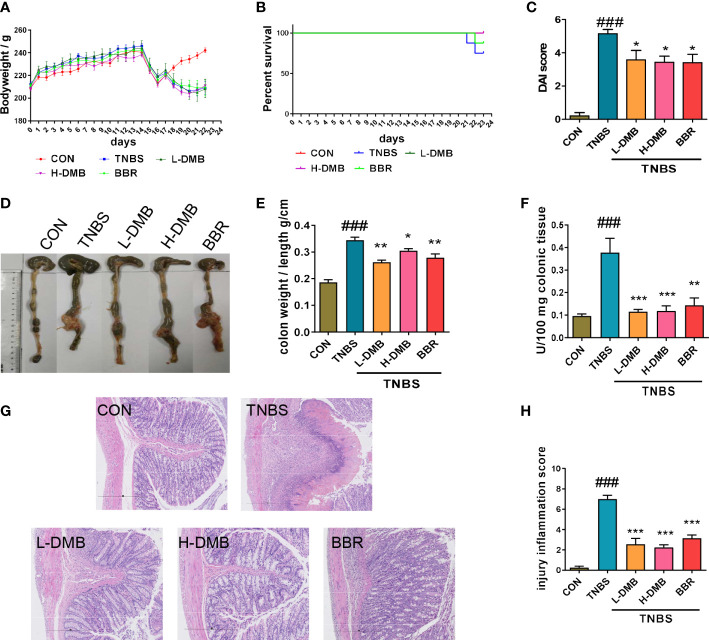
DMB relieved intestinal barrier injury by inhibiting TLR4 signaling in the experimental rat CD model. **(A)** The body weight of rats in the CD model. **(B)** The percentage survival of rats. **(C)** DAI scores of rats in different groups. **(D)** Macroscope of colonic tissues of rats in different groups. **(E)** Colon mass score marked with the ratio of colon weight and colon length. **(F)** The MPO activity in colonic tissues. **(G)** H&E staining of colonic tissues (100×) in CD rats. **(H)** Injury inflammation score of colonic tissue. Statistical analysis was performed using one-way ANOVA. *n* = 8. ###*p* < 0.005 (#: the TNBS group versus the CON group) and *p < 0.05, **p < 0.01, ***p < 0.005 (*: the DMB or BBR group versus the TNBS group).

The colitis rats induced by TNBS were successfully characterized by weight loss, DAI, and the rate of survival (**Figures 2A–C**). Notably, DMB significantly relieved TNBS-induced colitis in rats (**Figures 2A–C**). Colon atrophy, the colonic tissue mass score, neutrophil infiltration, and histological damage of rats were significantly relieved by DMB administration (**Figures 2D–H**). These results revealed that DMB relieved TNBS-induced colitis in rats.

### DMB inhibited TLR4 signaling in an MyD88-dependent and -independent manner

**Figure 3:**
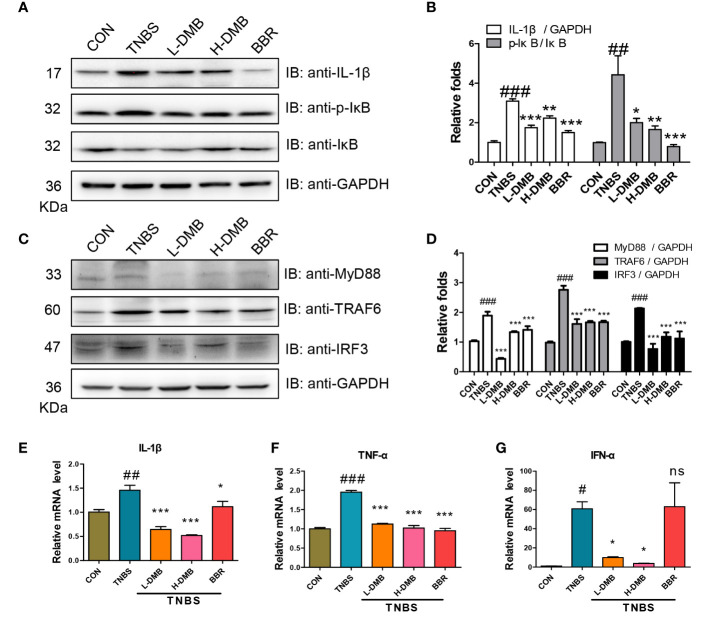
DMB inhibited TLR4 signaling in a MyD88-dependent and -independent manner. **(A)** The protein expressions were assessed by immunoblotting for IL-1β, IκB, and p-IκB (Ser32) in colonic tissue; GAPDH served as reference. **(B)** The grayscale analysis of IL-1β and the ratio of p-IκB/IκB. **(C)** The protein expression of MyD88, TRAF6, and IRF3 in colonic tissue; GAPDH served as reference. **(D)** The grayscale analysis of MyD88, TTRAF6, and IRF3. **(E–G)** The mRNA levels of IL-1β, TNF-α, and IFN-α in colonic tissues. Statistical analysis was performed using one-way ANOVA. *n* = 3. #*p* < 0.05, ##*p* < 0.01, ###*p* < 0.005 (#: TNBS group versus the CON group) and *p < 0.05, **p < 0.01, ***p < 0.005, ns, non-significantly (*: the DMB or BBR group versus the TNBS group).

Extracellular stimulus signals were intracellularly transduced by TLR4 signaling in an MyD88-dependent and -independent manner. Western blotting results showed that both the cleaved IL-1β and the phosphorylation of IκB were obviously increased in the TNBS group, prompting the idea that the TLR4 signaling was hyperactivated by TNBS (**Figures 3A, B**). DMB suppressed the phosphorylation of IκB and blocked the level of cleaved IL-1β (**Figures 3A, B**). Both key proteins TRAF6 and IRF3 were increased under TNBS stimulation and recovered by DMB and BBR treatment (**Figures 3C, D**). The effectors of IL-1β, TNF-α, and IFN-α were upregulated in the TNBS group and inhibited markedly by DMB and BBR treatment (**Figures 3E–G**).

Furthermore, RAW264.7 cells were stimulated by LPS to activate TLR4 signaling. The results showed that the protein of TRAF6 and IRF3 and the phosphorylation of IκB and cleaved IL-1β were also obviously increased in the LPS group (**Figures S1A–D**), but DMB and BBR inhibited TLR4 signaling-related proteins and downregulated the phosphorylation of IκB. The mRNA expressions of IL-1β, TNF-α, and IFN-α were also inhibited by DMB (**Figures S1E–G**). The above results revealed that DMB extensively inhibited TLR4 signaling in an MyD88-dependent or -independent manner *in vivo* and *in vitro*.

### DMB targeted MD-2 to inhibit the overactivation of TLR4 signaling

**Figure 4:**
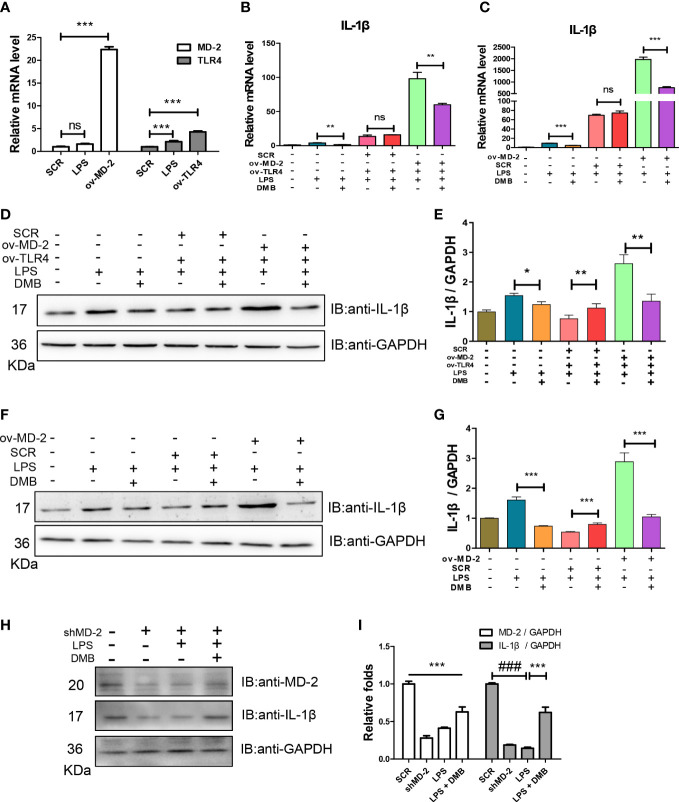
DMB blocked TLR4 signaling by targeting the MD-2 protein. **(A)** The mRNA levels of TLR4 and MD-2 after overexpression of TLR4 and MD-2, respectively. **(B)** The mRNA levels of IL-1β after co-overexpression of TLR4 and MD-2. **(C)** The mRNA levels of IL-1β after overexpression of MD-2. **(D)** The protein expressions of IL-1β after co-overexpression of TLR4 and MD-2. **(E)** The grayscale analysis of IL-1β after co-overexpression of TLR4 and MD-2. **(F)** The protein expressions of IL-1β after overexpression of MD-2. **(G)** The grayscale analysis of IL-1β after overexpression of MD-2. **(H)** The protein expressions of MD-2 and IL-1β after interfering with shMD-2. **(I)** The grayscale analysis of MD-2 and IL-1β after interfering with shMD-2. Statistical analysis was performed using one-way ANOVA. *n* = 3. ###*p* < 0.001 (#: the LPS group versus the SCR group); *p < 0.05, **p < 0.01, ***p < 0.001, ns: non-significantly (*: the DMB group versus the LPS group).

To further explore the molecular mechanism of DMB in inhibiting TLR4 signaling, the overexpression plasmids of TLR4 and MD-2 were matured and transfected into RAW264.7 cells (**Figure 4A**). The mature IL-1β was elevated after LPS stimulation when co-transfected with TLR4 and MD-2 plasmids, and DMB effectively inhibited the expression and maturation of IL-1β (**Figures 4B, D, E**). As shown in **Figures 4B, D**, overexpression of TLR4 alone did not cause the expression and maturation of IL-1β after LPS incubation, while the maturation of IL-1β was exacerbated by additional overexpression with MD-2, and the expression and maturation of IL-1β were inhibited by DMB (**Figures 4C, F, G**). The function of LPS in activating IL-1β was antagonized by interfering with MD-2. However, DMB even promoted the matured IL-1β level after knockdown of MD-2 (**Figures 4H, I**). These results suggested that DMB blocked the activation of TLR4 signaling induced by LPS *via* targeting the MD-2 protein.

### DMB interacted with phenylalanine at position 76 in the hydrophobic pocket of the MD-2 protein to inhibit the hyperactivation of TLR4 signaling

**Figure 5:**
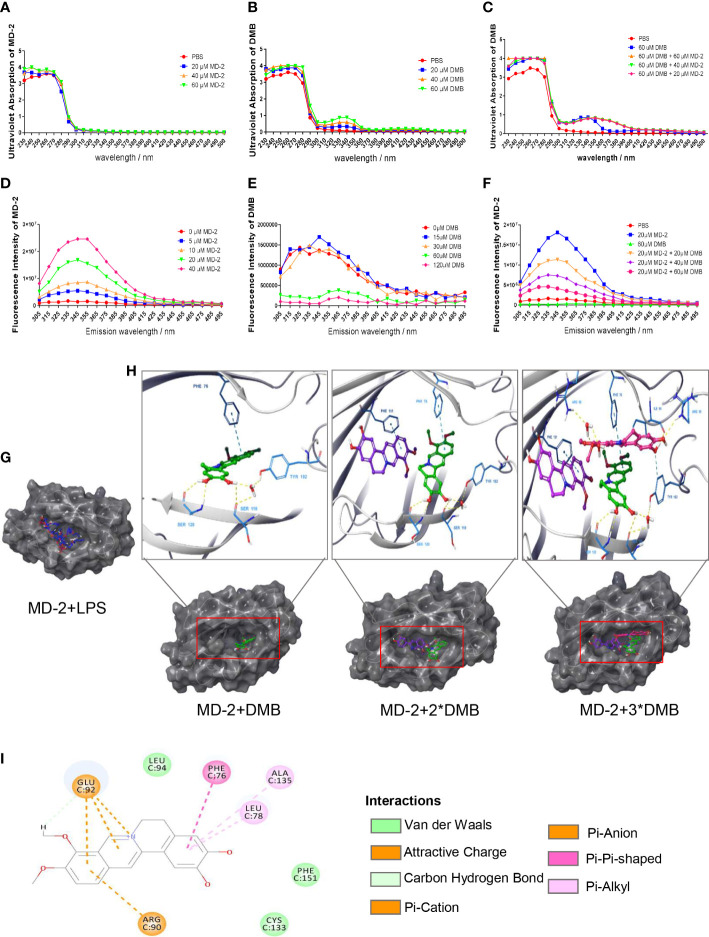
Spectroscopic analysis of the interaction between DMB and MD-2. **(A)** Ultraviolet absorption of MD-2 in different concentrations. **(B)** Ultraviolet absorption of DMB in different concentrations. **(C)** Ultraviolet absorption of DMB after co-incubation with MD-2 in different concentrations. **(D)** The fluorescence emission spectrum of MD-2 in different concentrations after excitation at 280 nm. **(E)** The fluorescence emission spectrum of DMB in different concentrations after excitation at 280 nm. **(F)** The fluorescence emission spectrum of MD-2 co-incubated with DMB in different concentrations after excitation at 280 nm. **(G)** Molecular docking of MD-2 and LPS. **(H)** Molecular docking of MD-2 and DMB (1:1; 1:2, and 1:3). **(I)** DMB interacted with the phenylalanine at position 76 of MD-2 in a pi–pi T-shaped interaction. *n* = 3.

Furthermore, the MD-2 protein was purified and renatured *in vitro* after expression in *E. coli BL21*. The results showed that the MD-2 protein has no characteristic UV absorption wavelength; however, the UV absorption wavelength of DMB was red-shifted after co-incubating with the MD-2 protein (**Figures 5A–C**). Based on aromatic amino acids, the MD-2 protein had the largest fluorescence emission wavelength at 345 nm (**Figure 5D**); the fluorescence emission wavelength of DMB was only 10% of MD-2 (**Figure 5E**). DMB reduced the fluorescence emission intensity of the MD-2 protein in a dose-dependent manner after excitation at 280 nm (**Figure 5F**). The results of molecular docking showed that both LPS and DMB entered the hydrophobic pocket of MD-2, and DMB interacted with the phenylalanine residue at position 76 of the MD-2 protein in a pi–pi T-shaped interaction to reduce the fluorescence emission intensity of MD-2 and promote the red shift of DMB UV absorption (**Figures 5G–I**).

**Figure 6:**
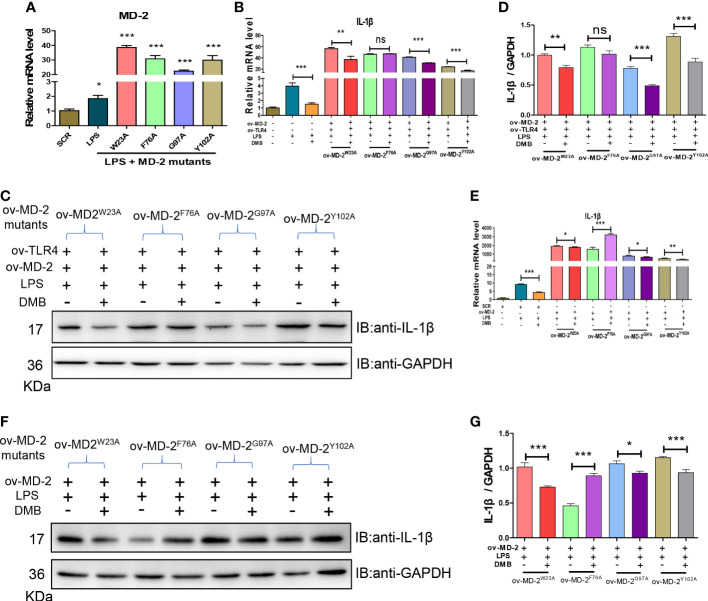
DMB interacted with phenylalanine at position 76 of the MD-2 protein. **(A)** The mRNA levels of MD-2 after overexpression of MD-2 mutants. **(B)** The mRNA levels of IL-1β after overexpression of MD-2 mutants and TLR4. **(C)** The protein expression of IL-1β after overexpression of MD-2 mutants and TLR4. **(D)** The grayscale analysis of IL-1β after overexpression of MD-2 mutants and TLR4. **(E)** The mRNA levels of IL-1β after overexpression of MD-2 mutations. **(F)** The protein expressions of IL-1β after overexpression of MD-2 mutations. **(G)** The grayscale analysis of IL-1β after overexpression of MD-2 mutations. Statistical analysis was performed using one-way ANOVA. *n* = 3. *p < 0.05, **p < 0.01, ***p < 0.001, ns, non-significantly (*: the DMB group versus the LPS group).

To find out the interaction site between DMB and MD-2, based on the molecular docking and the characteristic of MD-2 fluorescence excitation, four types of MD-2 mutant plasmids were constructed by site-directed mutagenesis, and transfected into RAW264.7 cells (**Figure 6A**). The mRNA and protein expression of IL-1β were elevated by LPS stimulation, and reversed by DMB treatment after co-expression of TLR4 and MD-2 mutants (**Figures 6B–D**), whereas DMB even enhanced the content of IL-1β at MD-2 ^F76A^ mutation after transfecting MD-2 mutants alone (**Figures 6E–G**). The UV absorption wavelength of DMB was also red-shifted after co-incubating with MD-2 mutations (**Figure S2A**), and four types of MD-2 mutants had different fluorescence emissions (**Figure S2B**). DMB reduced the fluorescence emission of MD-2 mutants by different degrees; the reduction of fluorescence intensity was the lowest in the MD-2^F76A^ mutant (**Figures S2C, D**). A direct comparison between all MD2 mutants and WT MD2 is shown in **Figure S3**. The results showed that the overexpression of MD-2 and its mutants was not changed by LPS stimulation or LPS plus DMB incubation. At the same time, the expression level of endogenous MD-2 is much lower than that of exogenous MD-2 in RAW264.7 cells (**Figures S3A–C**). These results revealed that DMB inhibited TLR4 signaling by interacting with phenylalanine at position 76 in the hydrophobic pocket of the MD-2 protein.

### DMB competitively blocked LPS binding to the MD-2 protein

**Figure 7:**
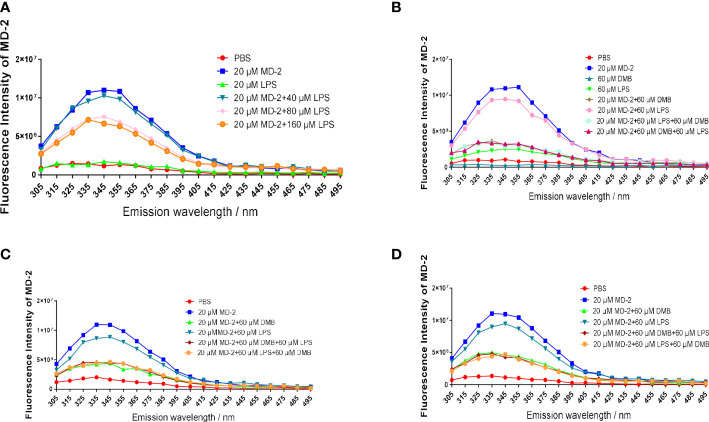
DMB competitively inhibited LPS binding to the MD-2 protein. **(A)** The fluorescence emission spectrum of the MD-2 protein co-incubated with LPS after excitation at 280 nm. **(B)** The fluorescence emission spectrum of the MD-2 protein co-incubated with LPS or DMB for 15 min, and then DMB or LPS was added for 5 min. **(C)** The fluorescence emission spectrum of the MD-2 protein co-incubated with LPS or DMB for 60 min, and then DMB or LPS was added for 5 min. **(D)** The fluorescence emission spectrum of the MD-2 protein co-incubated with LPS or DMB for 30 min, and then DMB or LPS was added for 30 min. *n* = 3.

To further compare the binding affinity of DMB and LPS to the MD-2 protein, MD-2 was co-incubated with DMB or LPS separately. The fluorescence emission intensity of the MD-2 protein was reduced by incubation of LPS in the lower dose range, whereas excessive addition of LPS did not continue to reduce the fluorescence emission intensity of MD-2 (**Figure 7A**). LPS was incubated with the MD-2 protein for 15 and 60 min, respectively, and DMB continued to reduce the fluorescence emission intensity of MD-2 within 5 min. However, when MD-2 was preferentially incubated with DMB for 15 and 60 min, LPS did not continue to decrease the fluorescence intensity of the MD-2 protein anymore within 5 min (**Figures 7B, C**). Moreover, DMB continued to reduce the fluorescence emission intensity of MD-2 within 30 min after being pre-incubated with LPS for 30 min (**Figure 7D**). These results revealed the affinity of DMB and the MD-2 protein, which was higher than that of LPS and the MD-2 protein.

### DMB rescued mice from LPS-induced acute sepsis by inhibiting TLR4 signaling

**Figure 8:**
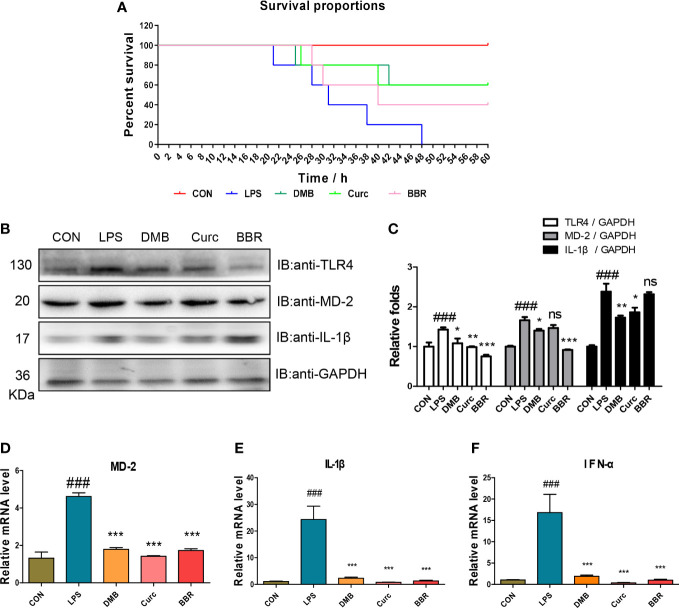
DMB ameliorated acute sepsis induced by LPS in mice. **(A)** The survival proportion of mice in different groups. **(B)** The protein expressions of TLR4, MD-2, and IL-1β in colonic tissue. **(C)** The grayscale analysis of TLR4, MD-2, and IL-1β in colonic tissue. **(D–F)** The mRNA level of MD-2, IL-1β, and IFN-α in colonic tissue. Statistical analysis was performed using one-way ANOVA. *n* = 6. ###*p* < 0.001 (#: the LPS group versus the CON group) *p < 0.05, **p < 0.01, ***p < 0.001, ns, non-significantly (*: the DMB, Curc, or BBR group versus the LPS group).

To further verify that DMB targeted MD-2 to inhibit TLR4 signaling, a mouse model of acute sepsis was constructed by injecting with LPS. All the mice in the LPS group died within 48 h, but DMB and Curc, a well-known MD-2 inhibitor (24), significantly increased the survival proportions of mice (**Figure 8A**). The protein expression of TLR4, MD-2, and IL-1β was significantly changed in colonic tissue (**Figures 8B, C**). The mRNA levels of MD-2, IL-1β, and IFN-α were significantly increased in the LPS group and were decreased by DMB, Curc, and BBR administration in liver and colonic tissue (**Figures 8D–F**). All these results showed that DMB rescued mice from LPS-induced acute sepsis by inhibiting TLR4–MD-2 signaling.

## Discussion

The intestinal innate immune system not only provides an effective immune barrier to resist the invasion of pathogenic microorganisms but also exerts immune response to PAMP (25). Correspondingly, hyperactivated inflammatory response and immune dysregulation promote intestinal permeability and intestinal mucosal damage in patients with colitis (26). In our previous study, we found that DMB inhibited the activation of TLR4 signaling and eliminated the accumulation of ROS in DSS-induced UC mice (18, 23). In this study, we further explored the pharmacological activity and molecular mechanism of DMB in the treatment of colitis induced by TNBS, and we revealed that DMB alleviated colitis and suppressed the activation of TLR4 signaling in an MyD88-dependent and -independent manner in TNBS-induced colitis rats and LPS-induced RAW264.7 cells. Based on the inhibitory effect of DMB on TLR4 signaling, we hypothesized that the target of DMB was between the upstream of TLR4 signaling and the cell membrane receptor complex. Therefore, the target and mechanism of DMB in the inflammatory response were explored in this study.

**Figure 9:**
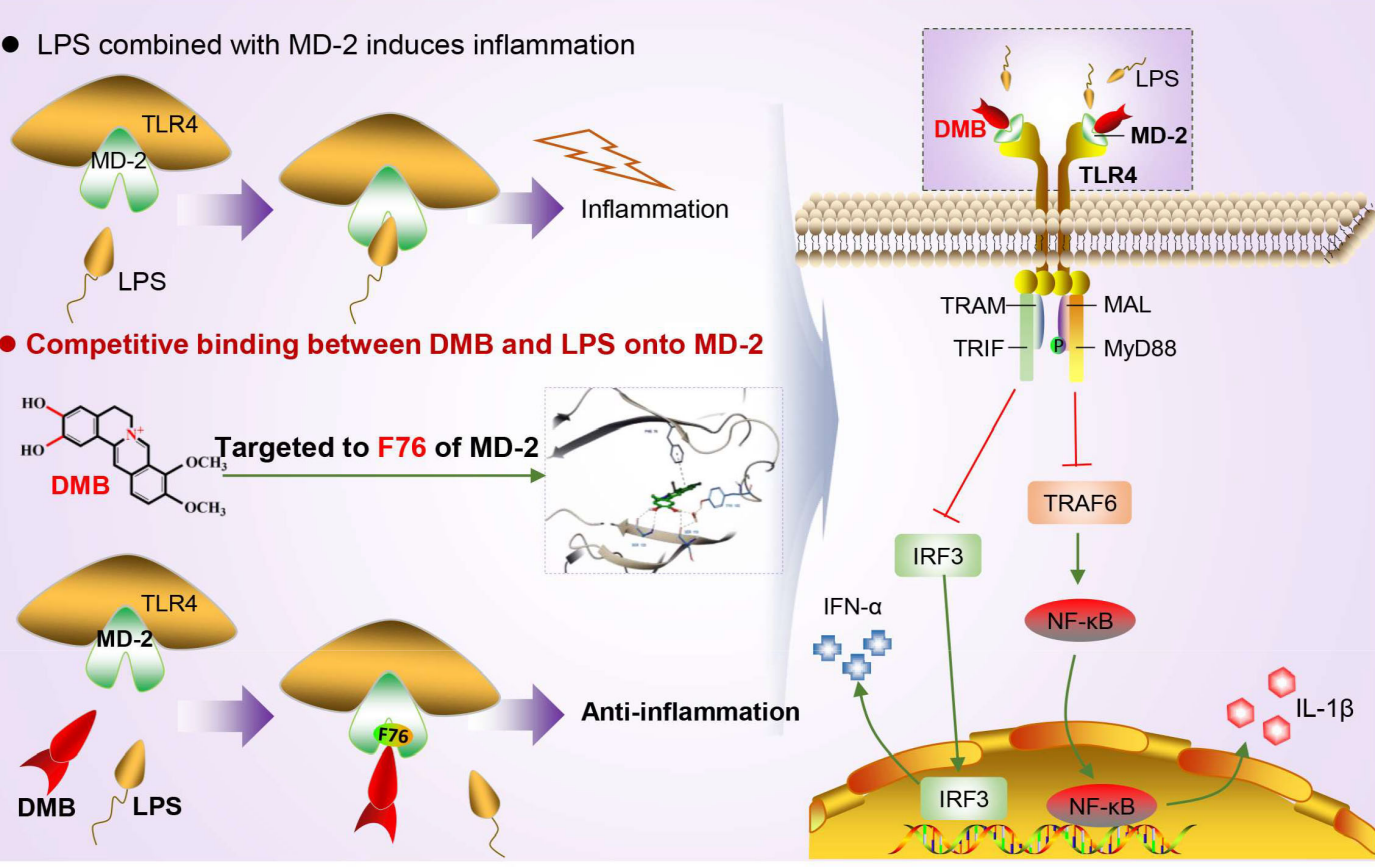
DMB targeted MD-2 to block inflammatory response by inhibiting the TLR4 signaling pathway. DMB significantly blocked TLR4 signaling in both an MyD88-dependent and an MyD88-independent manner *via* embedding into the hydrophobic pocket of the MD-2 protein with non-covalent bonding to phenylalanine at position 76 in a pi–pi T-shaped interaction.

Emerging evidence suggested that overexpression of TLR4 alone did not enhance the responsiveness of cells to LPS (15, 27). In this study, we also found that overexpression of TLR4 alone in RAW264.7 cells did not elevate the maturation of IL-1β induced by LPS. Conversely, the maturation of IL-1β was significantly promoted by the overexpression of MD-2 alone or co-overexpression with TLR4. Furthermore, knockdown of MD-2 by shRNA maintained hyporesponsiveness to LPS in RAW264.7 cells, and DMB no longer suppressed the maturation of IL-1β. However, DMB had a minor effect on IL-1β mRNA but a significant difference on matured IL-1β protein. These results also suggested that DMB might have another target in inhibiting IL-1β maturation; in our previous study, we found that DMB blocked the maturation of IL-1β in inflammation by inhibiting TLR4-mitochondria signaling (20). In this study, our results further revealed that the key amino acid residue that interacted with DMB was phenylalanine at position 76 of the MD-2 protein. The inhibitory effect of DMB on the TLR4 signaling pathway was abolished by the mutation of phenylalanine to alanine at position 76 of the MD-2 protein (**Figure 9**). Interestingly, DMB preferentially combined with the MD-2 hydrophobic pocket in a pi–pi T-shaped interaction and blocked the interaction of the MD-2 protein with LPS.

MD-2 plays an essential role in numerous inflammatory diseases and may be a potential therapeutic target for inflammation (28, 29). In the present study, we demonstrated that DMB was a reliable MD-2 inhibitor. To further verify that DMB targeted MD-2 to relieve inflammatory response, a murine sepsis model was used in our research. Curc was used as a positive control. The results revealed that the TLR4 signaling is hyperactivated by LPS and DMB inhibited excessive inflammatory response, inactivated TLR4 signaling in colonic tissue, and rescued mice from LPS shock, which confirmed that the potential target of DMB was the MD-2 protein. Additionally, DMB could inhibit the expression of MD-2 to eliminate inflammatory response in colonic tissue; this regulation might be related to the small molecular weight and hydrophobic property of DMB.

## Conclusion

DMB embedded in the MD-2 hydrophobic pocket in a pi–pi T-shaped interaction with phenylalanine at position 76 of MD-2 to block LPS to activate TLR4 signaling. Taken together, DMB is a promising inhibitor of the MD-2 protein to suppress the hyperactivated TLR4 signaling in inflammatory response.

## Data availability statement

The original contributions presented in the study are included in the article/**Supplementary Material**. Further inquiries can be directed to the corresponding author.

## Ethics statement

All animal care procedures in this study were carried out according to the Guide for the Care and Use of Laboratory Animals (Ministry of Science and Technology of China, 2006) and approved by the related ethical regulations of China Pharmaceutical University.

## Author contributions

YXZ: design of the study and drafting the article. PL: figures design, acquisition, and analysis of data. HL: analysis and interpretation of data. HJ: data collection. YX: acquisition of data. YQZ: acquisition of data. YBZ: conceptualization and obtained funding. RL: conception and design of the study and final approval of the version to be submitted. All authors contributed to the article and approved the submitted version.
