# Supplementary material for: Demethyleneberberine alleviated the inflammatory response by targeting MD-2 to inhibit the TLR4 signaling
Source: Front Immunol. 2023 Apr 24;14:1130404. doi: 10.3389/fimmu.2023.1130404 (PMC10165096; doi:10.3389/fimmu.2023.1130404)
Supplement: Supplementary file 1 [file DataSheet_1.docx]

**Supplementary figure legends**

**Supplementary figure 1**


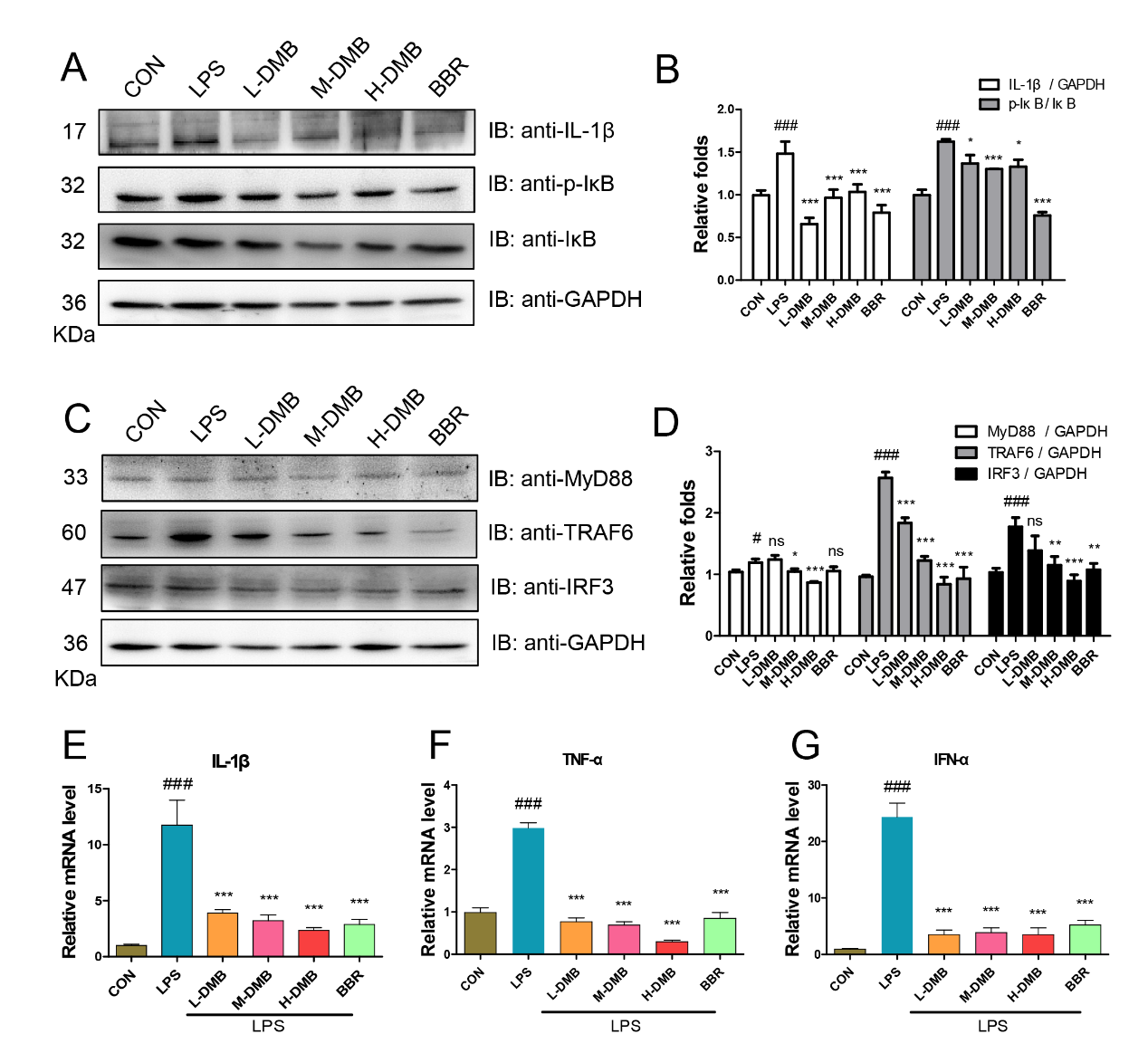


**Supplementary figure 1 DMB inhibited TLR4 signaling in MyD88-dependent and independent ways. (A)** The protein expressions of IL-1β, IκB and p-IκB in RAW264.7 cells, GAPDH was served as reference. **(B)** The grayscale analysis of IL-1β and p-IκB / IκB. **(C)** The protein expression of MyD88, TRAF6 and IRF3 in RAW264.7 cells, GAPDH was served as reference. **(D)** The grayscale analysis of MyD88, TTRAF6 and IRF3. **(E-G)** The mRNA levels of IL-1β, TNF-α and IFN-α in RAW264.7 cells. (L-DMB: 5 μM; M-DMB: 10 μM; H-DMB: 20 μM and BBR: 20 μM, respectively). Statistical analysis was performed using one-way ANOVA. n=3. #*P*<0.05, ###*P*<0.001 (#: LPS group versus the CON group); **P*<0.05, ***P*<0.01, ****P*<0.001, ns: non-significantly, (*: DMB or BBR group versus the LPS group).

**Supplementary figure 2**

**
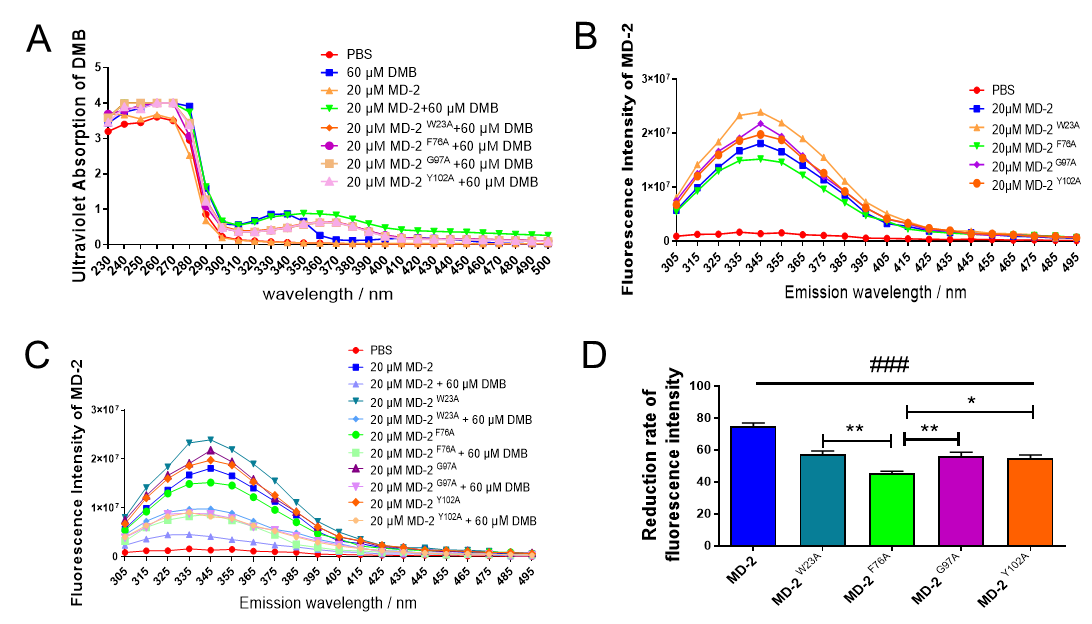
**

**Supplementary figure 2. Spectroscopic analysis of the interaction between DMB and MD-2 mutants. (A)** Ultraviolet absorption of DMB after co-incubating with MD-2mutants. **(B)** The fluorescence emission spectrum of MD-2 mutants after excitation at 280nm. **(C)** The fluorescence emission spectrum of MD-2 mutants co-incubated with DMB after excitation at 280nm. **(D)** The reduction rate of fluorescence emission intensity after co-incubating with MD-2 mutants.

**Supplementary figure 3**


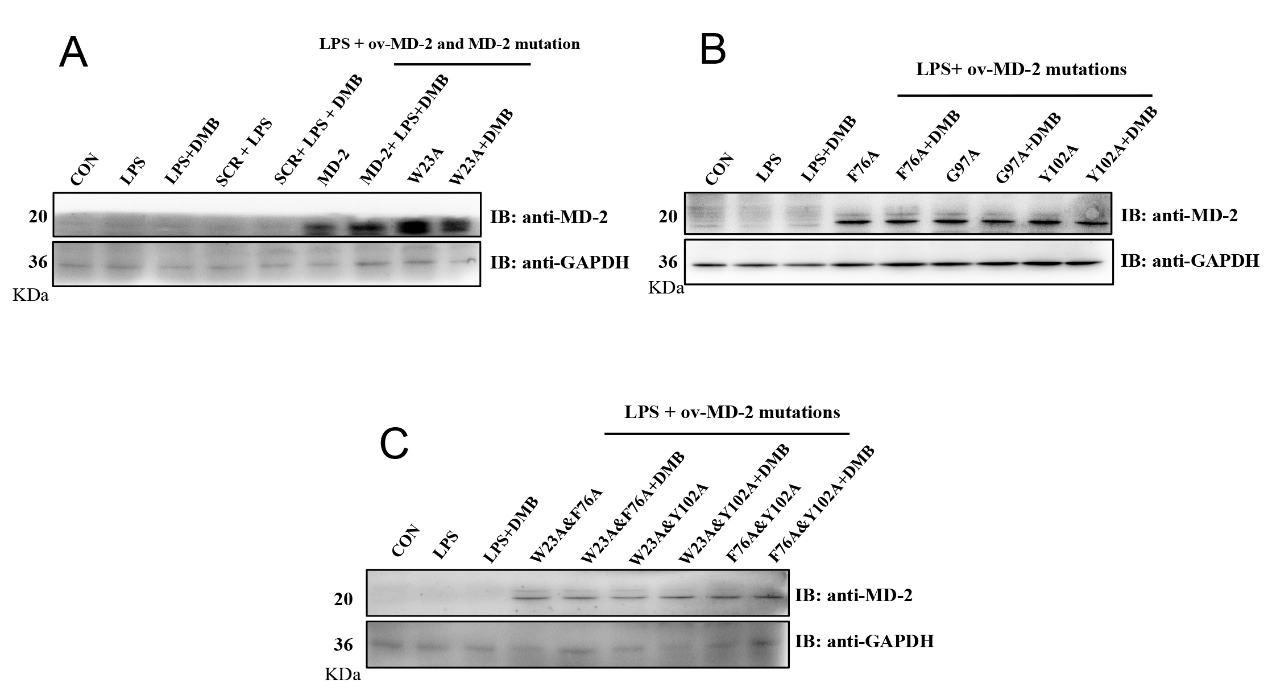


**Supplementary figure 2.** T**he protein expressions of WT MD-2 and its mutants in RAW264.7 cells. (A-C)** The protein expressions of endogenous and exogenous MD-2 or its mutants by LPS stimulation or LPS plus DMB incubation in RAW264.7 cells.
